# Some Further Considerations of the Subject of Dentition

**Published:** 1893-01

**Authors:** C. N. Peirce


### SOME FURTHER CONSIDERATIONS OF THE SUBJECT OF DENTITION.

BY C. N. PEIRCE, D.D.S.

An editorial in the December International Dental Journal can most assuredly be sustained and supplemented by every dental practitioner of twenty years' experience. In that period he must have had innumerable cases of trifacial disturbance which could have had, apparently, no other origin but tooth-development. How any one who has been for years in the general practice of medicine, with even a very limited experience in pediatrics, can for one moment doubt the disturbing influence of what is very properly termed "interrupted dentition" is a marvel to those who understand the origin and mode, or process, of development of the teeth and appreciate their physiological and pathological relations to the entire system.

That we have in this locality a degree of nervous endowment by or through the terminal distribution of the hypersensitive trifacial every anatomist attests, and that these terminæ are the seat of disordered sensibility and function from abnormal local, as well as systemic disturbances, every pathologist will also affirm.

To show the close relation existing between these parts, in their progressive developmental changes, and general systemic disturbances, we have to bring to mind the influence of disturbed nutrition upon the structures themselves. In the diseases of infancy and childhood, when severe and protracted, how unmistakably does the influence of disturbed nutrition, through imperfect calcification, draw its unfailing lines across the labial or buccal surfaces of the incisors, cuspids, bicuspids, or molars, the teeth so affected varying with the age at the time of the health-disturbance.

The genius presiding over the function of nutrition writes in such indelible characters that one who is at all familiar with the periods of calcification or progressive solidification of tooth-tissues, can with accuracy give the date of the beginning and the ending of the systemic derangement. The histologist, microscopist, and biologist, all unite in establishing the data of tooth-development, and give us the seventh week of intra uterine life, when the embryo is but little over an inch in length, as the time for the development of the enamel-germ or matrix, to be followed in the ninth week by the dentine-germ, these from that date progressing in their development until the seventeenth week, when the incisors and cuspids on the zonal- or border-line between these two germs receive depositions of the salts of lime. The odontoblasts on the upper border of the dentine germ are converted into dentine, while the lower ends of the enamel-cells, or ameloblasts,—adamantoblasts of Tomes,—are converted into enamel. By the end of the nineteenth week the same developmental processes have reached the molars, and from this period until the fortieth week, or birth, the integration and calcification of these deciduous tooth-germs progress simultaneously, so that at this period (birth) the eight incisors have their crowns quite complete, while the four cuspids and four first molars are fully one-half formed, and the four second molars about one-fourth or one-third calcified. Now, let us suppose that during the period of gestation the mother has been in a retrograde condition, so far as health is concerned, suffering, if you please, with phthisis, or any other constitutional condition interfering seriously with nutrition; what chance have these erupting, deciduous teeth for permanency or durability? Let us examine the child with such parentage at thirty months of age; what do we find? twenty teeth in position, with rarely more than two, and probably not any, well covered with enamel. If they are not defective on the cutting edges and masticating surfaces, the vertical surfaces will be pitted and broken, uncalcified or imperfectly calcified enamel-cells, predisposing the teeth to dental decay and resulting in premature loss. And this from improper nutrition during embryonic life.

Let us now follow the life of the child with the influence of *its health* on the development of the permanent teeth. As early as the sixteenth week of intra-uterine life we have originating the enamel-germ of the four first permanent molars, and the twenty permanent teeth which are to succeed the twenty deciduous. These enamel-germs are quickly followed by preparation for the dentine-germs. Progressive developmental stages in all of these prepare them for the commencement of calcification by birth or shortly thereafter. By the end of the first year calcification has commenced and progressed to a greater or less extent in the four first molars and the four central incisors. During this period, and before it is sixteen months old, but beyond the eighth month, the child is suffering from what may very appropriately be termed interrupted dentition, to the extent of convulsions at intervals of days, or it may be weeks, but continuing in this enfeebled condition for four months or longer. What will be the condition of these eight permanent teeth when erupted? As they make their appearance through the gum, the horizontal or masticating surfaces of the molars and the cutting edges of the incisors will be imperfect in their enamel covering. Let us now suppose that tooth-development has progressed uninterruptedly until the child is five or five and a half years old, and it then becomes ill with scarlet-fever, diphtheria, or other serious disturbance,—by this time the second permanent molar has progressed from the period of its origin, three months after birth, until calcification has commenced at five years on its masticating surface,—where now does malnutrition write its history? On the crowns of the five anterior teeth, near the gum-line, or neck, and the masticating surfaces of the second permanent molars. If the systemic disturbance occurs still later in life, between the ages of eight years and twelve, then the crowns of the third molars, or wisdom-teeth, will in all probability show the result of malnutrition. Such is the history of systemic disturbances—improper or defective nutrition—upon the developing teeth.

If the teeth are responsive to the extent we have shown to systemic disturbances, it demonstrates most certainly a very close relationship with, and dependence upon, a normal arterial and nervous system. This relationship once established, it is certainly safe to assume that they, with their close connection with the terminal branches of the sensory, as well as the motor branches of the great hypersensitive trigeminus, may play sad havoc with their manifold functions. The great argument against interference on behalf of tooth-eruption is that "dentition is a physiological process." Undoubtedly it is; but not more so than hundreds of other processes. Yet how often is the specialist, aurist, ophthalmologist, obstetrician, etc., called in to aid in what should be a normal physiological process?

But it is with facts we have to deal, and they are so numerous and well-authenticated that it ought to be supererogation to recite them, and it certainly would be if medical and dental practitioners would thoroughly inform themselves on the local causes or conditions which may induce these grave, sympathetic, nervous disturbances which interfere with the function of organs, near or remote. While it should not be held responsible for all abnormities or pathological complications to which infancy, childhood, or youth are liable, yet, appreciating its possibilities, it is unsafe to ignore it while either deciduous or permanent tooth-development is in progress.

Purely normal dentition depends upon a complete correspondence between the development of tooth-germ and its calcification at its base, or growing extremity, and the absorption, or other wise removal of the overlying structures, so that, as the soft structures are solidified, the previously-formed and dense part of the tooth will be lifted from its base, and thus prevent impingement upon the uncalcified germ or dentine papilla. It is the backward pressure upon the uncalcified germ which produces constitutional disturbance. When this is indicated the lancet should be thoroughly used, that for the time the obstruction to the advancing crown may be entirely removed.

A few instances under the observation and treatment of the writer will suffice. Infants who for forty-eight hours or longer time have not had quiet sleep or retained food, have, on liberating the advancing tooth with the lancet, gone at once to sleep and remained for six or eight hours, with abatement of all unfavorable symptoms. Also those suffering from catarrh, with threatened pneumonia, have, with a similar operation, been entirely relieved. Boys of fourteen years of age affected by persistent gum over second permanent molar, and suffering from retention of deciduous molars, may be relieved by lancing the gum in the one case and removing the deciduous molars in the other. Also severe facial neuralgia from impacted cuspids and third molars has time and again been relieved by the removal of the offending tooth.

These few cases are abundantly sufficient to illustrate the susceptibility of the trigeminus to iritation with expressions in localities receiving the same, though quite remote from the cause, and also by the persistence of trouble to serious systemic or constitutional disturbances.

